# Unveiling Morphological Diversity: An Anatomical Investigation of the Foramen Transversarium in the Cervical Vertebrae

**DOI:** 10.7759/cureus.67143

**Published:** 2024-08-18

**Authors:** Deepa G, Ratna Prabha J, Mrudula Chandrupatla, Kusneniwar G N, Shrikrishna B H

**Affiliations:** 1 Anatomy, All India Institute of Medical Sciences, Bibinagar, Bibinagar, IND; 2 Anatomy, Navodaya Medical College, Raichur, IND; 3 Community Medicine and Family Medicine, All India Institute of Medical Sciences, Bibinagar, Bibinagar, IND; 4 Otorhinolaryngology and Head and Neck Surgery, All India Institute of Medical Sciences, Bibinagar, Bibinagar, IND

**Keywords:** anatomical, variations, morphometry, foramen transversarium, cervical vertebrae

## Abstract

Purpose: This study aimed to investigate the morphological variations in the foramen transversarium of the cervical vertebrae and their clinical implications. Understanding these variations is crucial for accurate diagnosis, treatment planning, and surgical procedures involving the cervical spine.

Materials and methods: This descriptive cross-sectional study was conducted at the AIl India Institute of Medical Sciences, Bibinagar, India, and involved 150 dry cervical vertebrae specimens. Measurements of the anteroposterior and transverse dimensions, as well as anatomical variations such as accessory foramina and bilateral symmetry, were recorded using vernier calipers.

Results: Out of 150 vertebrae, 149 had foramina on both sides, while one had a single foramen on the right. The anteroposterior diameter ranged from 1.0 to 10.0 mm on the right (mean: 5.13 ± 1.25 mm) and 2.0 to 8.5 mm on the left (mean: 5.08 ± 1.11 mm). The transverse diameter ranged from 2.0 to 9.0 mm on the right (mean: 5.54 ± 1.06 mm) and 2.0 to 8.0 mm on the left (mean: 5.42 ± 1.07 mm). Statistical analysis indicated symmetry in morphological dimensions. The morphological variations included unilateral and bilateral accessory foramina, incomplete accessory foramina, and asymmetrical foramina. Circular shapes were predominant (76% on the right, 75% on the left), with other shapes, such as oval and irregular shapes, being less common.

Conclusion: These findings enhance the understanding of cervical spine anatomy, aiding in the interpretation of radiographic images and the planning of surgical procedures. This research highlights the need for precise anatomical knowledge to improve patient outcomes in cervical spine-related interventions.

## Introduction

The foramen transversarium (FT), a crucial anatomical feature of the cervical vertebrae, has attracted significant attention due to its morphological complexity and clinical implications. Research has emphasized the importance of comprehending the variations in the FT, such as the existence of accessory foramina, morphometric variances, and anatomical differences, as they are of paramount clinical significance for orthopedic surgeons, neurosurgeons, radiologists, and other healthcare professionals [1-4]. Therefore, revealing the morphological intricacies of the FT is imperative for advancing clinical practices and ensuring optimal patient care. By focusing on the detailed analysis of the FT, this research aimed to uncover new insights that could have implications for clinical practice, surgical procedures, diagnostic imaging, and therapeutic interventions.

This study aimed to investigate the morphological variations in the FT and their clinical implications. The rationale for this study lies in the significance of understanding the dimensions, variations, and anatomical features of the FT for clinical practice. Knowledge of these morphometric aspects is crucial for clinicians because it can aid in accurate diagnosis, treatment planning, and surgical procedures related to vertebrobasilar insufficiency, spinal decompression, and other cervical spine conditions [2,3].

The FT plays a significant role in the passage of important structures, such as the vertebral artery, vein, and nerve branches. Variations in the morphology of the FT, including the accessory foramina, absence of coastal elements, and different shapes, can have clinical implications and may impact the interpretation of radiographic images and CT scans [5-7]. Understanding these variations is essential for avoiding misdiagnosis, planning interventions, and ensuring the safety and efficacy of surgical procedures in the cervical region.

Moreover, the study of the FT is not only relevant for clinical practice but also contributes to the broader understanding of cervical spine anatomy. By elucidating the morphological complexities of the FT, this research can enhance the existing knowledge base in anatomy and provide valuable insights for future studies in the field [8,9].

In conclusion, investigating the morphological complexity of the FT in the cervical vertebrae is essential for advancing clinical practice, improving diagnostic accuracy, and enhancing surgical outcomes. This study provides valuable insights that can benefit various medical specialties and contribute to understanding cervical spine anatomy.

## Materials and methods

This descriptive cross-sectional study was conducted at the Department of Anatomy, All India Institute of Medical Sciences (AIIMS), Bibinagar, India. The research started with approval from the Institutional Research Committee. The study was exempted from the Institutional Ethics Committee because the samples included dry cervical vertebrae. This study aimed to examine the morphological diversity of the FT in the cervical vertebrae. The sample size included 150 dry cervical vertebra specimens. Vertebrae with evidence of traumatic injury affecting the integrity of the FT and those with congenital abnormalities were excluded from the study, as these factors may confound the interpretation of morphological variations specific to the research focus.

The specimens were measured using a vernier caliper. We used vernier calipers, first ensuring their calibration by setting them to zero and verifying accuracy against a standard reference. Specimens were positioned on a stable platform, and the anteroposterior and transverse diameters were measured using the internal and external jaws of the calipers, respectively. Each measurement was repeated three times to ensure precision, and the average values were recorded. The anteroposterior and transverse dimensions, shape, and anatomical variations of the FT, such as the presence of accessory foramina and bilateral symmetry, were recorded. The collected data was entered into a Microsoft Excel spreadsheet.

Descriptive statistics were obtained by calculating the means, standard deviations, ranges, and percentages to describe morphological variations in the FT. Graphs, charts, and diagrams were used to present data effectively.

## Results

**Figure 1:**
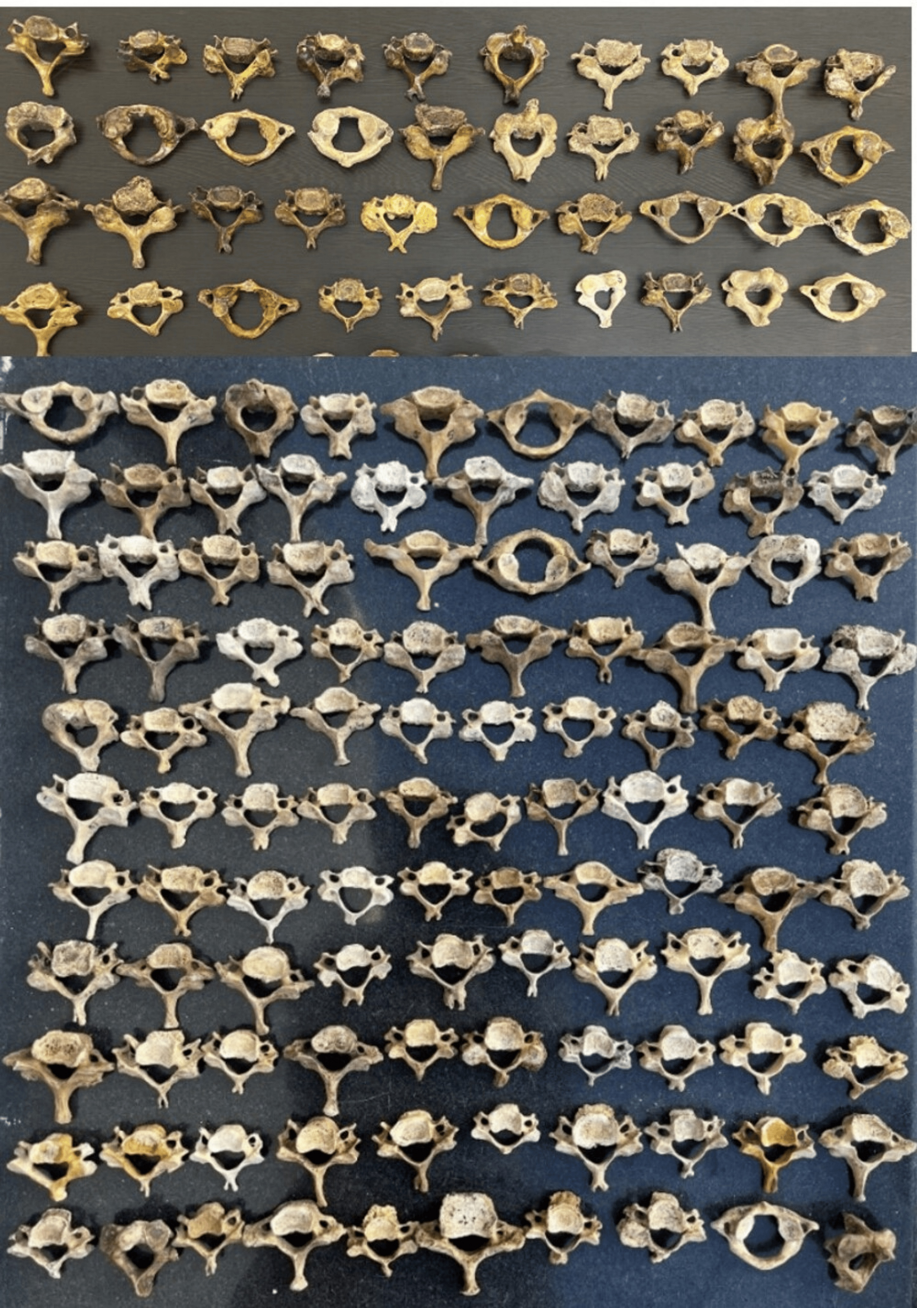
Total number of cervical bones used for the study

**Figure 2:**
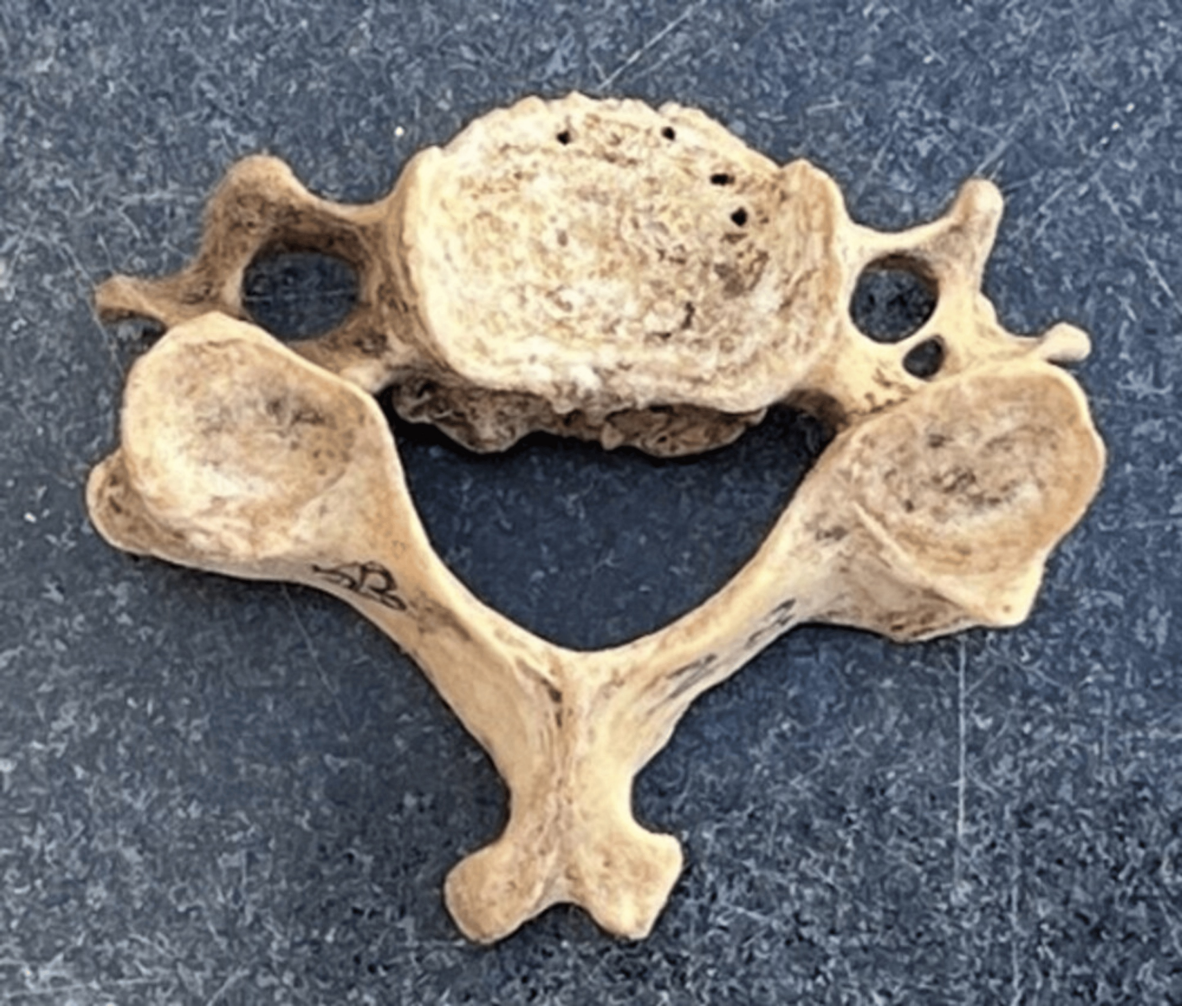
Left-sided accessory foramen transversarium

**Figure 3:**
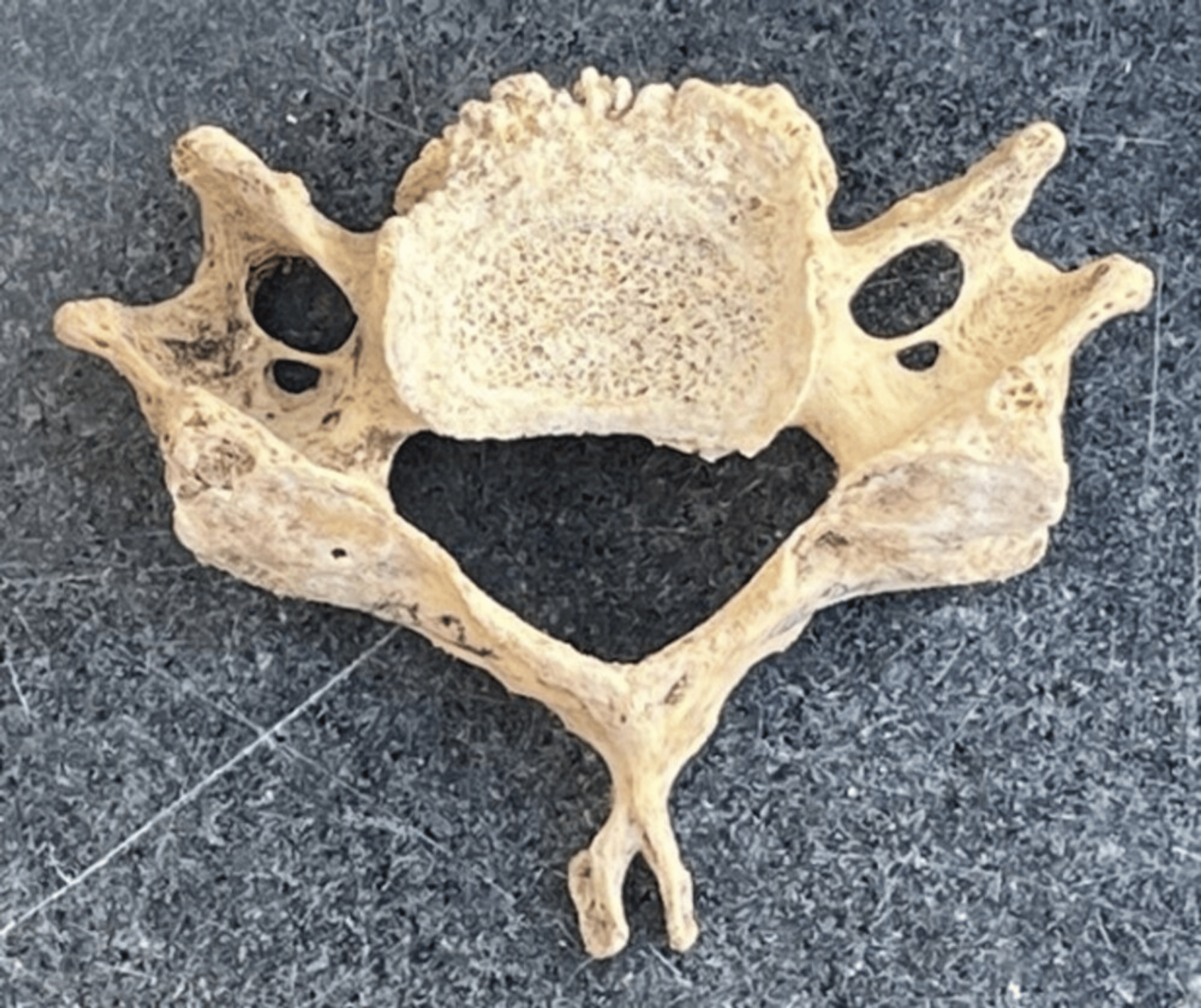
Bilateral accessory foramen transversarium

**Figure 4:**
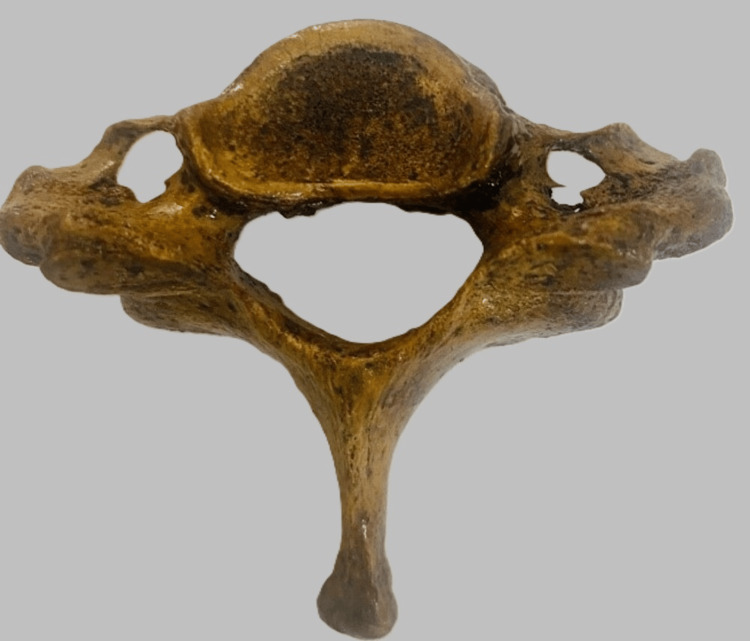
Bilateral incomplete accessory foramen transversarium

**Figure 5:**
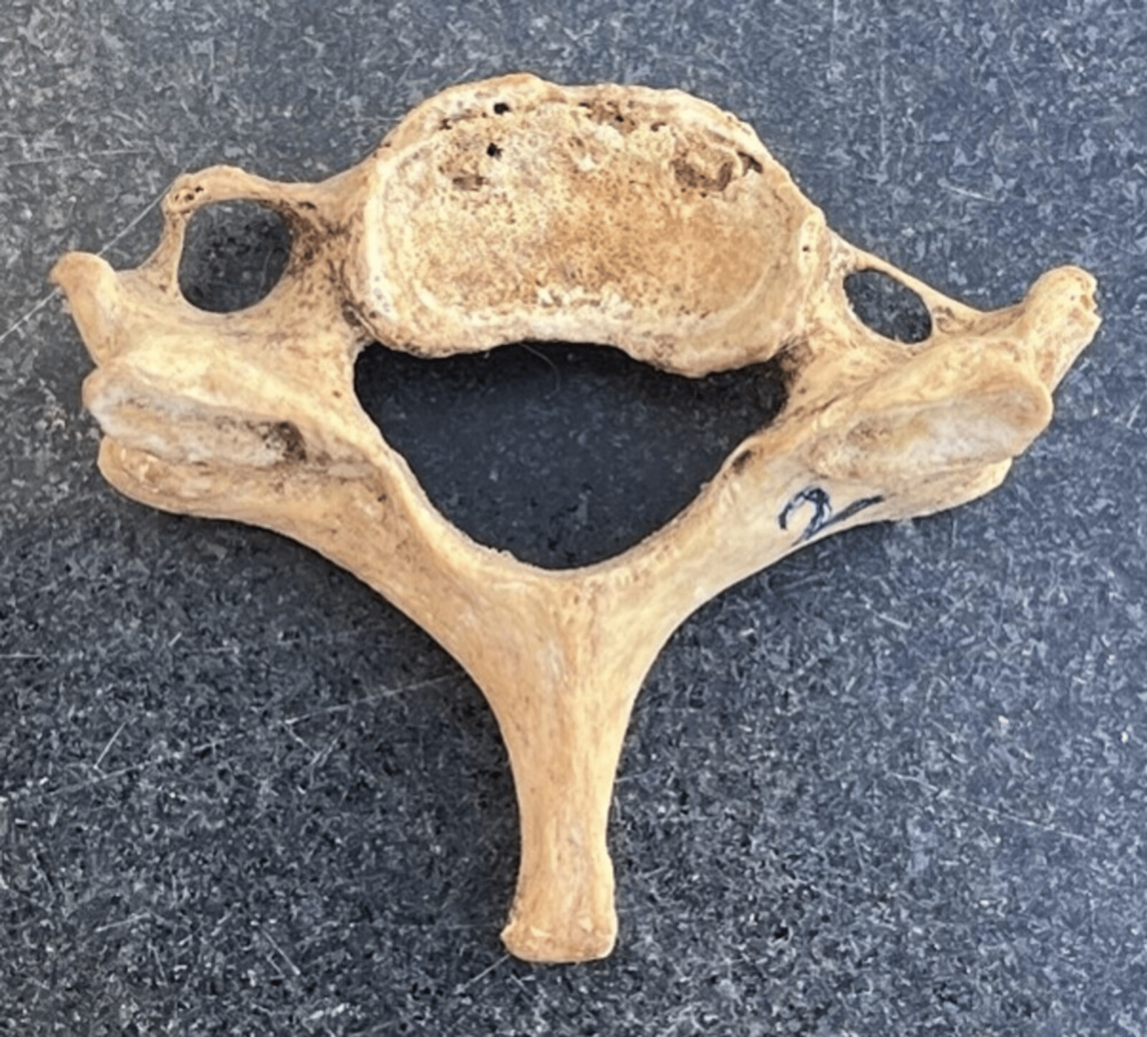
Asymmetric foramen transversarium of both sides

Out of the 150 cervical vertebrae (Figure 1) included in the study, 149 had an FT on both sides and 1 cervical vertebra had a single FT (on the right side). Various morphological variations (Figures 2-5) were identified among the samples collected.

**Figure 6:**
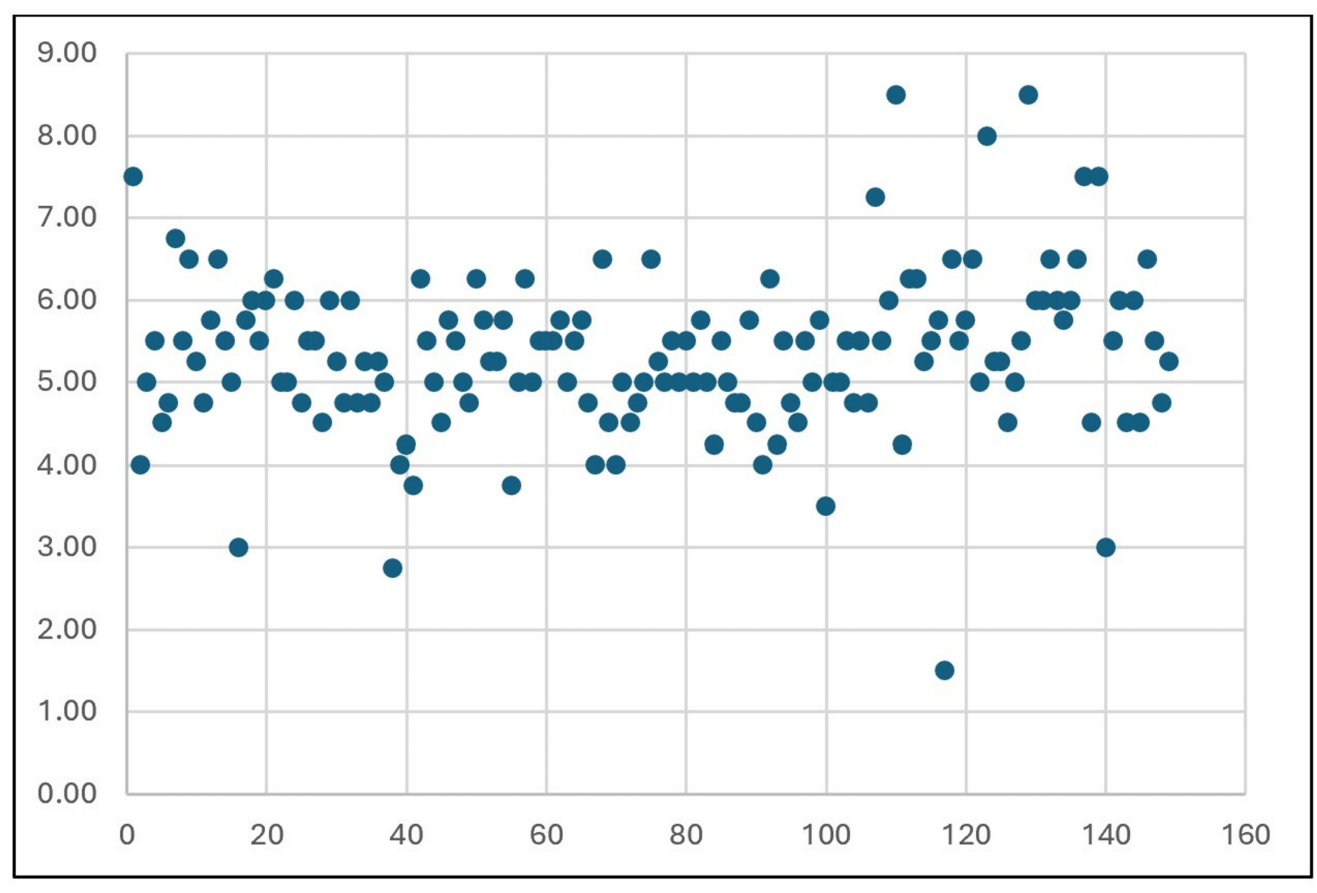
Scatter plot of the mean diameters of the right foramen transversarium

**Figure 7:**
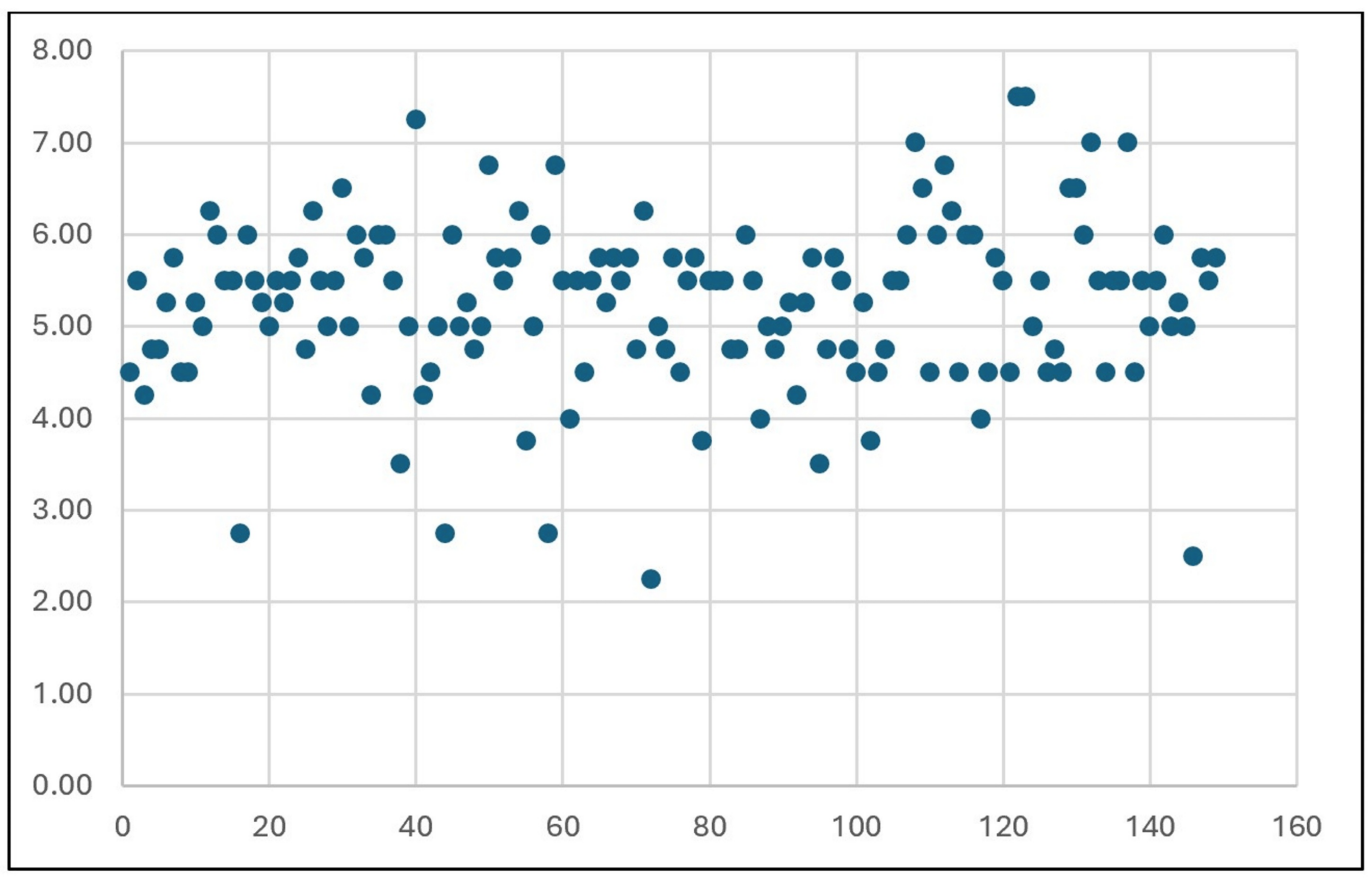
Scatter plot of the mean diameters of the left foramen transversarium

This study examined the dimensions of the FT (Table 1) in 150 dry cervical vertebrae. The anteroposterior diameter ranged from 1.0 to 10.0 mm on the right (mean: 5.13 ± 1.25 mm) and 2.0 to 8.5 mm on the left (mean: 5.08 ± 1.11 mm). The transverse diameter ranged from 2.0 to 9.0 mm on the right (mean: 5.54 ± 1.06 mm) and 2.0 to 8.0 mm on the left (mean: 5.42 ± 1.07 mm). The mean diameter ranged from 1.5 to 8.5 mm on the right (mean: 5.33 ± 0.97 mm) and 2.25 to 7.5 mm on the left (mean: 5.25 ± 0.92 mm). The mean diameters of both the right and left FT were represented through a scatter plot (Figures 6, 7).

**Table 1:** Dimensions of the foramen transversarium of the cervical vertebrae FT: foramen transversarium

Dimensions of FT		Range	Mean ± SD
Anteroposterior diameter	Right (N = 150)	1.0-10.0	5.13 ± 1.25
	Left (N = 149)	2.0-8.5	5.08 ± 1.11
Transverse diameter	Right (N = 150)	2.0-9.0	5.54 ± 1.06
	Left (N = 149)	2.0-8.0	5.42 ± 1.07
Mean diameter	Right (N = 150)	1.5-8.5	5.33 ± 0.97
	Left (N = 149)	2.25-7.5	5.25 ± 0.92

Paired mean differences between the right and left sides (Table 2) were analyzed for anteroposterior, transverse, and mean diameters. The anteroposterior diameter had a mean difference of 0.043 mm (p = 0.627), the transverse diameter had a mean difference of 0.120 mm (p = 0.266), and the mean diameter had a mean difference of 0.082 mm (p = 0.355). None of these differences were statistically significant, indicating symmetry between the right and left FT in the measured dimensions.

**Table 2:** Paired mean differences in the anteroposterior, transverse, and mean diameters of the right and left foramen transversarium SD: standard deviation; CI: confidence interval; t: t-test, df: degrees of freedom; Sig: significance

Diameter	Mean difference	SD	95% CI	t	df	Sig
R-L anteroposterior diameter	0.043	1.095	-0.13-0.22	0.486	148	0.627
R-L transverse diameter	0.120	1.319	-0.09-0.33	1.117	148	0.266
R-L mean diameter	0.082	1.081	-0.09-0.25	0.928	148	0.355

There was no significant difference between the transverse diameter (mean difference: 0.12, t-test: 1.11, df: 148, p-value: 0.266), anteroposterior diameter (mean difference: 0.043, t-test: 0.48, df: 148, p-value: 0.627), or mean diameter (mean difference: 0.082, t-test: 0.928, df: 148, p-value: 0.355) of the right and left FT. The differences were not statistically significant.

**Figure 8:**
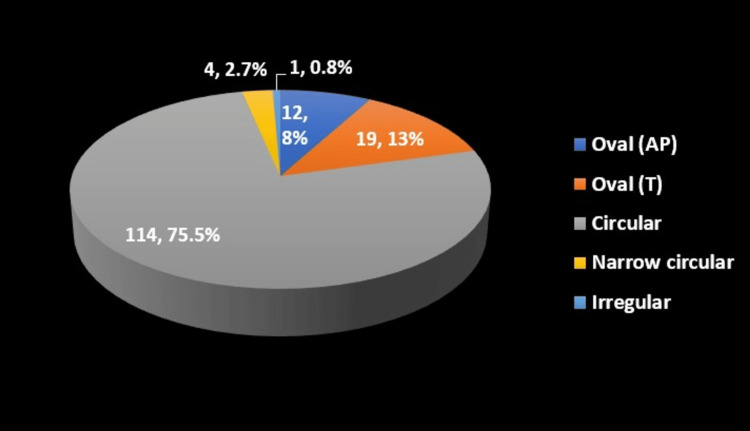
Pie-graph showing the shape of the foramen transversarium on the right side AP: axes perpendicular; T: tangential

**Figure 9:**
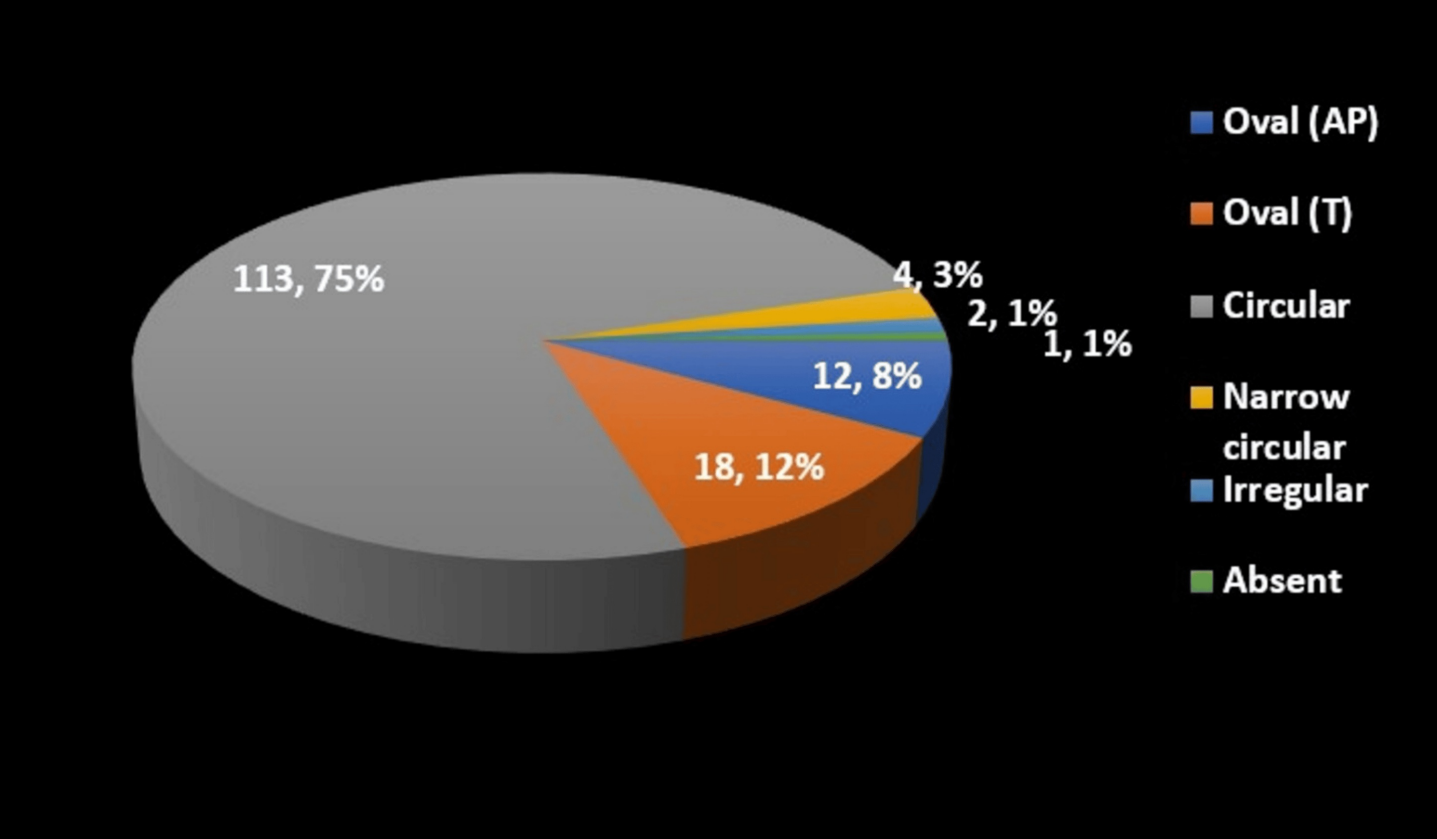
Pie-graph showing the shape of the foramen transversarium on the left side AP: axes perpendicular; T: tangential

Our study also examined the morphological diversity of the FT. The shape of the FT on the right side (Table 3) was predominantly circular in 114 specimens (75.5%), with other shapes, such as oval anteroposteriorly (8%), oval transversely (13%), narrow circular (2.7%), and irregular (0.8%), being less common. On the left side (Table 4), the shape distribution was similar, with 113 specimens (75%) being circular, and other shapes included oval anteroposterior (8%), oval transversely (12%), narrow circular (3%), irregular (1%), and one instance of absence (1%). These findings are shown in Figures 8, 9.

**Table 3:** Shape of the foramen transversarium on the right side FT: foramen transversarium; AP: axes perpendicular; T: tangential

Shape of FT right	Frequency	Percentage (%)
Oval (AP)	12	8%
Oval (T)	19	13%
Circular	114	75.5%
Narrow circular	4	2.7%
Irregular	1	0.8%
Total	150	100%

**Table 4:** Shape of the Foramen transversarium on the left side FT: foramen transversarium; AP: axes perpendicular; T: tangential

Shape of FT left	Frequency	Percentage (%)
Oval (AP)	12	8%
Oval (T)	18	12%
Circular	113	75%
Narrow circular	4	3%
Irregular	2	1%
Absent	1	1%
Total	150	100%

Additionally, morphological variations in the FT (Table 5) included unilateral accessory FT in eight specimens (5.3%), bilateral accessory FT in seven specimens (4.7%), unilateral incomplete accessory FT on the right and left sides in two specimens each (1.3%), bilateral incomplete accessory FT in one specimen (0.7%), and asymmetrical FT on both sides in six specimens (4%).

**Table 5:** Morphological variations in the FT FT: foramen transversarium

Morphological variation	Frequency	%
Unilateral accessory FT	8	5.3%
Bilateral accessory FT	7	4.7%
Unilateral incomplete accessory FT (right)	2	1.3%
Unilateral incomplete accessory FT (left)	2	1.3%
Bilateral incomplete accessory FT	1	0.7%
Asymmetrical FT of both sides	6	4%

These findings indicate a high prevalence of circular FT shapes and a notable presence of accessory and incomplete accessory FT variations, highlighting the anatomical diversity of the foramen transversarium in cervical vertebrae.

## Discussion

The FT in the cervical vertebrae is responsible for transmitting the vertebral artery, vein, and sympathetic plexus. The present study aimed to elucidate the morphological diversity of the FT across the cervical vertebrae (C1-C7). Our findings contribute to the existing knowledge base on this critical anatomical structure and its potential clinical implications.

In the present study, the shape of the FT varied across the samples analyzed. Circular shapes are the most common, making up 76% of the total. Oval shapes, both with long axes perpendicular (AP) and tangential (T) to the main axis, are present in 21% of the samples combined. Narrow circular shapes account for a smaller portion (3%), while irregular shapes are rarely reported in only one case (Tables 3-4). The findings in our study indicate a high prevalence of circular FT shapes and a notable presence of accessory and incomplete accessory FT variations, highlighting the anatomical diversity of the FT in cervical vertebrae. This system offers a standardized approach for describing FT variations, facilitating future research and clinical applications. Notably, the observed prevalence of each type (circular being the most common) suggests a potential population-specific pattern.

Variations in the FT have been extensively studied, revealing the presence of accessory transverse foramina in addition to the main foramen [1]. In our study, morphological variations, including unilateral accessory FT (Figure 2), were observed in eight cervical vertebrae and bilateral accessory FT (Figure 3) was observed in seven cases. We also reported that cervical vertebrae with incomplete accessory FT (Figure 4) were observed in 2% of the samples, and asymmetrical FT of both sides (Figure 5) was observed in six bone samples (Table 5). Anatomical studies focusing on the incidence of accessory foramina in different populations have provided valuable insights into the prevalence of these variations and their clinical implications [10]. The identification of the accessory foramina and the morphometric analysis of the FT contribute to a better understanding of the anatomical diversity of the cervical vertebrae [11]. Additionally, case reports highlighting rare occurrences, such as the absence of the FT in the atlas vertebra, shed light on the spectrum of anatomical variations that can be encountered [12]. Hence, understanding the incidence and morphological characteristics of these accessory foramina is essential for the accurate diagnosis and treatment of cervical spine pathologies. Surgical considerations for these variations include careful identification of the FT during procedures, especially in cases where the accessory foramina is present.

Imaging techniques such as CT angiography have proven invaluable in visualizing the vertebral arteries within the FT, aiding in surgical decision-making, and ensuring patient safety [13]. Furthermore, studies have shown that variations in the FT are common in imaging modalities such as magnetic resonance imaging (MRI) and CT, emphasizing the need for a comprehensive understanding of these anatomical structures [14]. Future studies employing larger sample sizes and advanced imaging techniques such as multidetector computed tomography (MDCT) could provide a more comprehensive picture of FT morphology.

The detailed morphological and morphometric variations in the FT, as highlighted in various studies, underscore the importance of precise anatomical knowledge for medical professionals dealing with cervical spine pathologies [15].

The presence of the vertebral arteries within the FT, particularly at the cervicothoracic junction, poses challenges and considerations for surgical interventions in this region [16]. Understanding the variations in the origin and course of the vertebral artery as it enters the FT is crucial for avoiding potential complications during surgical procedures [17]. Moreover, the unique anatomy of the C7 vertebra, with its distinct pedicles, laminae, and FT, necessitates a tailored approach to surgical planning [18].

The observed variations in FT morphology could be attributed to various developmental factors. Additionally, mechanical stress during fetal development or early life might influence FT formation. Further research is necessary to elucidate the precise interplay of these factors in shaping the FT morphology.

The FT serves as a vital passage for the vertebral artery and sympathetic plexus, playing a critical role in blood supply and autonomic nervous system function to the head and neck region. Variations in FT morphology can potentially influence the course and diameter of these neurovascular structures.

Our findings on FT morphology hold relevance for various clinical scenarios. A thorough understanding of FT anatomy helps avoid inadvertent injury to the vertebral artery or compromised blood flow.

Cadaveric studies have provided valuable insights into the morphological diversity of the FT, emphasizing the need for thorough anatomical training for healthcare professionals. Continued research in this field is crucial for advancing our understanding of these variations and improving patient outcomes in surgical interventions involving the cervical spine.

This study investigating the FT had several limitations. The small sample size and cross-sectional design restrict the generalizability of the findings. Additionally, the lack of clinical correlation prevents the direct linking of morphological variations to patient outcomes. Also, categorizing and comparing the morphological variations and dimensions of the foramina relative to their specific vertebral levels could not be done. Due to limitations in the sample size of specific cervical vertebrae, we were unable to perform a detailed individual comparison of the transverse foramina across C1 to C7. We suggest that future research with larger sample sizes could address these specific comparisons in more detail. The study could be improved by increasing the sample size and including clinical data.

## Conclusions

In conclusion, the present study has shed light on the remarkable morphological diversity of the FT in the cervical vertebrae. Our findings contribute to a growing body of knowledge concerning this crucial anatomical structure and its potential clinical implications. Further research exploring the developmental basis, evolutionary context, and biomechanical consequences of FT variations with larger, diverse samples and advanced imaging techniques is warranted to fully understand the clinical significance of FT morphology and its potential impact on neurovascular structures in the cervical region.
